# Mother and Infant

**Published:** 1880-12

**Authors:** 


[From "Hall's Family Doctor, or Health at Home."]

### MOTHER AND INFANT.

Of every two children born into the world, one dies before ten years have passed away.

Of every three children born, one dies before five years.

Of every five children born, one dies within a year.

With intelligent care, instead of half of all the children who come into the world dying within ten years, four-fifths of them ought to live, and would live.

That so many die is owing to the fact, in part, that mothers do not know soon enough that anything is the matter with their children, and the time is past for them to be saved; but they can know, they ought to know; and it is proposed here to show the mother how to know promptly that her child is not well, and to determine at once what part of the body suffers or is threatened.

If an infant is well its tongue is white, its eyes are bright, its flesh plump and full, and its skin soft and cool; the breathing is regular and easy and natural; when awake it is lively, cheerful, always disposed to laugh, always pleased to be played with; and, when asleep, it rests quietly, the countenance is composed, and conveys an expression of happy enjoyment.

### SIGNS OF DISEASE.

1. If the brow is contracted, there is pain in the head; if the head is hot, and turned restlessly from side to side, and the eyes stare, or there is a glare in them, there is inflammation, and

### WATER ON THE BRAIN

is threatened. Relief must be promptly had or the child is doomed. Put cold compresses or ice-pads on the head, and keep them there; compel the feet to keep warm, give a warm sitz-bath, and keep at these until the symptoms have abated, and the child sleeps quietly, or is disposed to eat or play.

2. If the lips are apart, with a kind of gritting, there is pain in the belly, and most certainly it has been fed too much or too often.

3. If the nostrils are drawn upward and there is quick breathing, there is pain in the chest; something is the matter with the lungs.

4. If there is a squinting in the eye, or bluish tint about the lips, and a kind of rotating movement of the eyeballs, convulsions will soon follow; there is indigestion, and a warm water emetic must be resorted to.

5. If the eyes are unusually dull, or there is an unnatural quickness, with a pearly look of the whites, brain-disease is approaching; give an enema and a dose of castor oil, and feed with regularity. [*Instead of an enema, use "Nelaton's Suppositories for Children." They are more convenient, act with sufficient promptness, and allay irritation instead of causing it.—Ed.*]

6. If a child, usually sprightly, holding itself up straight, is noticed to drop the head and seem languid and sleepy, or if it usually goes about from chair to chair, or is disposed to climb, but suddenly shows no disposition to do anything but lie down on the floor, there is something wrong in the stomach or bowels.

7. If there is crying and the legs are drawn up, there is indigestion, and the bowels are disordered.

8. In health, a child seldom carries the hands above the mouth; if that is observed repeatedly, there is something wrong in the head; the feet are cold, and they must be kept warm; if the bowels have not acted within ten hours, give an enema or a teaspoonful of castor-oil every hour until there is an action.

9. A healthy child, especially if not over two years old, is often carrying the hand to the mouth; but if it stops at the throat, croup is most likely forming; notice instantly if the feet or hands are cold, and turn to the article on croup.

10. In the first months of infancy, if the little one is well, it nurses, plays awhile, and then falls into a gentle, easy, good sleep; if it does not, if it is restless, especially if it starts up in its sleep, or wakes and whines, there is disturbance in the brain, and it should be seen to that the bowels are regular, feet warm, and food given at proper intervals, and of a suitable quality.

11. The first passages of an infant are dark colored, called the *meconium;* to bring this away is essential; if this is not done the child will suffer. But the first milk secreted, called the *colustrum*, acts as a purgative and carries the *meconium* before it; but if it does not come away oil must be given; sometimes warm water will answer; and in first confinements no milk appears sometimes for several days, hence any uneasiness of the first-born for the first few days may be caused by costiveness.

12. In health, young children go to sleep at once, and sleep quietly and soundly; if they are not well they do not lie down willingly at the regular hour for sleep, nor do they fall asleep at once, nor do they sleep continuously; there are frequent turnings and changings and wakings or startings up, often in alarm—then the bowels or head are out of order.

13. The passages of a healthy child are yellowish, and thicker than thick syrup, and are of uniform appearance, from three to four times a day; less than two is costiveness. It should be rectified with an enema or castor oil. More than three or four, and as thin as milk and light colored, show diarrhea, and are rapidly debilitating. Keep the belly warm, especially the feet and hands. Do not feed at oftener than five hours' interval, and let the food be boiled rice, sago, tapioca, exclusively, with a little boiled milk, until there is a reduction in frequency, and greater consistency is manifested. If the stools are curdy, or green, or smell badly, or come out with considerable force, there is disease to be treated as just named.

14. Crying: Young children never cry if all is well. If an infant cries, it is suffering. Each mother should notice the different cries of her child, for they mean different things; a cry from hunger is very different from a cry from hurt. A sticking pin causes a quick, instantaneous cry; a string or fastening which is too tight causes a fret at first, gradually increasing as the blood accumulates. The hunger cry does not come on suddenly, for the little thing begins to turn its head or face about, or makes motions with the tongue or lips; if it cannot find the breast it begins to make a noise, gets more and more impatient, and finally breaks out into a fierce, mad cry. The wisdom of the mother, then, should be called into requisition in deciding what cries mean, but in all cases attend to them; in a young infant it often means that a change of position is needed, or that it is too warm or too cold or thirsty, and wants a drink of cold water. A good plan always is when a child is fretful, notice at once if the feet and hands are warm. If the children are regularly fed as advised elsewhere they will never cry from hunger, unless their food is not sufficiently nourishing. A tearless cry means pain or suffering. When tears are abundant it is the cry of anger, and should always be disregarded; very young children will soon find out as to such crying, "It's no use knocking at the door any more."

A moaning cry always indicates suffering, and should never be neglected.

Breathing in a healthy child is regular, slow, easy, and full; in proportion as it is different in any case there is disease. If the breathing is loud, or labored, or quick, or fitful there is something wrong about the throat or lungs.

15. Cough of any kind, from the slightest hack to a deep or suppressed hoarseness, should always command immediate attention, from its uniform connection with croup, which almost always comes on in the evening, seldom all at once ; generally for several days previous there have been slight symptoms of a cold, increasing every day, but an occasional cough ; the face a little flushed, a little more excitability than common ; fitful temper, now a laugh, then a fret ; it may be measles or it may be croup ; notice the feet at once, and if there is any dampness in the clothing, inquire particularly about the exposures of the day preceding ; the questions to the nurse cannot be too minute or searching ; require specific, decided answers. Usually an attack of croup comes on after the child has been asleep a while, and it is likely to wake with a cough, different from an ordinary one ; it has a ring or whistle, or wheezing with it, a dry cough, no loosening of phlegm is present. This first cough may not wake the child, but do not leave it for an instant ; and if the second cough occurs, especially if the hand is carried to the throat, it is croup. Act accordingly.

But let it be deeply impressed on the mother's mind that if the child is kept comfortably warm for every second of its existence, especially the hands and feet; if it is fed at stated hours only, and has regular and abundant sleep, three-fourths of all the diseases of infancy and childhood will be swept from existence.

